# Pleomorphic Liposarcoma Unraveled: Investigating Histopathological and Immunohistochemical Markers for Tailored Diagnosis and Therapeutic Innovations

**DOI:** 10.3390/medicina60060950

**Published:** 2024-06-07

**Authors:** Ana-Maria Ciongariu, Dana-Antonia Țăpoi, Adrian-Vasile Dumitru, Adrian Bejenariu, Andrei Marin, Mariana Costache

**Affiliations:** 1 Department of Pathology, “Carol Davila’’ University of Medicine and Pharmacy, 020021 Bucharest, Romania; ana-maria.ciongariu@drd.umfcd.ro (A.-M.C.); dana-antonia.tapoi@drd.umfcd.ro (D.-A.Ț.); mariana.costache@umfcd.ro (M.C.); 2 Department of Pathology, University Emergency Hospital, 050098 Bucharest, Romania; adrian.bejenariu@rez.umfcd.ro; 3 Department of Plastic Surgery, “Carol Davila’’ University of Medicine and Pharmacy, 020021 Bucharest, Romania; andrei.marin@umfcd.ro

**Keywords:** liposarcoma, pleomorphic liposarcoma, immunohistochemistry

## Abstract

Liposarcomas are some of the most challenging soft tissue tumors and are subclassified into multiple subtypes with special histologic and molecular features. The peculiarities of each histopathological subtype influence the clinical behavior, management, and treatment of these neoplasms. For instance, well-differentiated liposarcomas are common soft tissue malignancies and usually display a favorable outcome. On the other hand, pleomorphic liposarcoma is the rarest, yet the most aggressive subtype of liposarcoma. This histopathological diagnosis may be challenging due to the scarce available data and because pleomorphic liposarcomas can mimic other pleomorphic sarcomas or other neoplasms of dissimilar differentiation. Nevertheless, the correct diagnosis of pleomorphic liposarcoma is of utmost importance as such patients are prone to develop local recurrences and metastases. Treatment usually consists of surgical excision along with radiotherapy and follow-up of the patients. Therefore, this review aims to assess the complex clinical, histological, and immunohistochemical features of liposarcomas in order to establish how these characteristics influence the management and prognosis of the patients, emphasizing the particularities of pleomorphic liposarcoma.

## 1. Introduction

Liposarcomas are rare soft tissue tumors regarded as a heterogeneous group comprising entities with distinct histopathological, immunohistochemical, and molecular features [1]. Liposarcomas are mainly subclassified into five subtypes with different morphologic and behavioral spectrum [1,2,3]. Well-differentiated liposarcoma and dedifferentiated liposarcoma stand for the largest subgroup of liposarcomas and exhibit a propensity for local recurrence and metastases [2,4]. Well-differentiated liposarcoma (atypical lipomatous tumor) is considered one of the most common soft tissue tumors, mainly affecting the extremities and retroperitoneum [5,6]. Superficial lesions are usually located in the subcutaneous fat and are diagnosed as atypical lipomatous tumors [7]. They exhibit locally aggressive behavior and a higher frequency of recurrence compared to conventional lipomas [8]. Upon microscopic examination, WDL typically consists of mature adipocytes of variable size, encompassed by fibrous stroma exhibiting atypical spindle cells with hyperchromatic nuclei [9]. Well-differentiated liposarcoma is related to dedifferentiated liposarcoma, a high-grade proliferation associated with similar genetic abnormalities, consisting of MDM2 (murine double minute 2) and CDK4 (cyclin-dependent kinase 4) amplification [10]. Although the histopathological diagnosis of such lesions is usually straightforward, distinguishing them from other entities can be difficult, especially in tumors with peculiar locations [10,11]. Immunohistochemical study and genetic analysis are the most useful methods of establishing the diagnosis [4]. Well-differentiated liposarcoma usually shows MDM2 and CDK4 expression [10,11,12]. However, some cases with atypical morphology and immunophenotype require FISH for identification of MDM2 amplifications, in order to avoid misdiagnosis [12,13,14,15,16,17].

Dedifferentiated liposarcoma is an uncommon neoplasm arising predominantly in the retroperitoneum and deep soft tissue of the extremities [4,18,19]. This malignancy is associated with an important risk of local recurrence, but its metastatic potential is related to the anatomic site of the tumor proliferation, as retroperitoneal masses have a worse prognosis [4]. The histopathological aspect of dedifferentiated liposarcoma usually consists of a lipogenic tumor proliferation with well-differentiated liposarcoma features, exhibiting an abrupt transition towards a non-lipogenic high-grade sarcoma [20].

The genetic background of dedifferentiated liposarcoma is characterized by MDM2 and CDK4 amplification; therefore, immunohistochemical expression of the corresponding markers is used for diagnosis [20]. A mutant TP53 immunophenotype, with hyperexpression of p53, is also reported in some studies [20,21]. Molecular analysis of this tumor is important, as new therapeutic targets have been identified [22]. Treatment of dedifferentiated liposarcoma also consists of surgical resection; therefore, the location of the lesion and evaluation of surgical resection margins have prognostic significance [20,23]. Tumors of the retroperitoneum are associated with a higher risk of recurrence, due to the difficulty of achieving complete surgical resection [23,24,25].

Dedifferentiated liposarcoma of the extremities is an uncommon entity with distinct clinical–pathological behavior and incompletely understood pathogenesis [20,25,26,27,28,29,30]. The most important entity that should be considered as a differential diagnosis for dedifferentiated liposarcoma of the extremities is myxoid liposarcoma [20,28].

Myxoid liposarcoma is a distinct entity with unique genetic and molecular features consisting of FUS-DDIT3 or EWSR1-DDIT3 fusion, typically occurring in younger patients [31]. Although myxoid liposarcoma displays a classic histopathological aspect, with lipogenic areas and basophilic stroma, tumors can exhibit unusual cellular features or metaplasia [32,33,34]. Consequently, the differential diagnosis should imply an analysis of DDIT3 mutation, especially in high-grade lesions [35,36]. This tumor presents variable histomorphology and peculiarities regarding therapeutic management and overall prognosis [36,37]. The role of FUS-DDIT3 oncoprotein in adipocytic neoplasia has been investigated in the context of ATP-dependent chromatin remodeling and alteration of genomic architecture, resulting in upregulation of tumorigenic pathway in cell lines of myxoid liposarcoma [38]. Researchers acknowledge that transcription factors, such as fusion oncoproteins, can activate oncogenic gene loci and bind to the BAF (barrier-to-autointegration factor) complex surface [38]. Further studies on the FUS-DDIT3 fusion oncogene in myxoid liposarcoma suggest a link between this mutation and JAK-stat signaling in the stem cancerous cell [38,39,40]. Currently, the most efficient treatment comprises surgical resection with preoperative radiotherapy frequently administered, whereas high-risk lesions of the limbs or trunk may receive chemotherapy [41,42]. However, in the advanced setting, new strategies, including targeted therapy, have been developed [43]. Considering the increasing number of studies on genetic alterations related to soft tissue sarcoma, histology-specific treatment protocols have been increasingly implemented and systemic treatment options apply for various liposarcoma subtypes [42,43].

Pleomorphic liposarcoma is a rare aggressive subtype accounting for 10% of all liposarcomas and it is diagnosed upon detection of multivacuolated pleomorphic lipoblasts upon microscopic examination [44]. This malignancy has no specific immunohistochemical or molecular features; therefore, diagnostic challenges include identifying lipoblasts that may be scarce and distinction from other pleomorphic sarcomas with special morphologic variants [44,45]. Myxoid pleomorphic liposarcoma is a recently defined neoplasm affecting young patients, disclosing mixed histological features and complex chromosomal alterations [46]. Although its distinctive clinical features support a separate classification of this tumor, studies suggest a link between it and conventional pleomorphic liposarcoma [46,47]. Considering the rarity of pleomorphic liposarcoma and its mimickers, we have reviewed the literature to gain further knowledge about this malignancy, emphasizing its histopathological and molecular features in correlation with its clinical behavior (Table 1).

## 2. Materials and Methods

This is a narrative review of the scientific literature. We included complete-length papers, using the PubMed search engine, focusing on liposarcomas and their morphological and immunohistochemical features, in correlation with prognosis and treatment. In order to gain further knowledge about pleomorphic liposarcoma, we investigated all the articles published between 2018 and 2023. We interrogated all types of English-language articles, comprising original studies, case reports, and reviews. The associated papers were searched for additional useful references. The research keywords were liposarcoma, dedifferentiated liposarcoma, myxoid liposarcoma, pleomorphic liposarcoma, and immunohistochemistry. The research papers were provided by four reviewers (A.M.C., D.A.Ț., A.B., and A.M.). Three reviewers (A.M.C., A.V.D, and M.C.) analyzed the articles on pleomorphic liposarcoma for information concerning clinical, radiologic, histopathological, immunohistochemical, and molecular features. The whole process was supervised and validated by two reviewers (A.V.D and M.C.)

## 3. Results

Pleomorphic liposarcoma is the rarest liposarcoma variant and is diagnosed based on the detection of multivacuolated pleomorphic lipoblasts within the specimens, while molecular characteristics of this tumor have constantly been reviewed and discussed, especially considering the new trends in novel investigations and therapeutic strategies [48,49]. Pleomorphic liposarcoma usually arises within deep soft tissue of the extremities of adult patients and is often a diagnostic challenge [49]. Wakely et al. reviewed their experience with fine needle aspiration as a diagnostic method for this tumor, focusing on the recognition of pleomorphic lipoblasts, which may be found in variable numbers [49]. The study included 20 patients with a 2.3/1 male/female ratio and a mean age of 58 and aspirates from the thigh, upper extremity, axilla, neck, and mediastinum were analyzed [49]. In most of the cases, examination of the specimens noted pleomorphic, epithelioid, and bizarre cells, while pleomorphic lipoblasts were absent or rare in 45% of the cases [49]. The researchers acknowledge that FNA biopsy may not be able to capture pleomorphic lipoblasts, due to the heterogeneous structure of this neoplasm [49]. Additionally, diagnosing pleomorphic liposarcoma and distinguishing it from other sarcomas are difficult, as ancillary studies may be of limited use [48,49]. The recent literature encompasses several studies on the genomics of each liposarcoma variant and suggests that pleomorphic liposarcoma differs from other subtypes, as it harbors chromosomal imbalances with large numbers of gains and deletions, but with no specific cytogenetic abnormality [50]. Tyler et al. examined the P53 deletion rate, by performing microarray analysis, noting the present mutation within 60% of samples [50,51]. Pleomorphic liposarcoma is associated with a poor prognosis and high recurrence, and its treatment is controversial [52]. Wang et al. reported a series of six patients with confirmed pleomorphic liposarcoma who underwent surgical excision of the tumor [52]. Researchers noted that five out of six patients developed local recurrences, with the shortest post-operative recurrence time of 4 months and the longest of 29 months [52]. Pleomorphic liposarcoma with peculiar locations has also been discussed throughout scientific literature, to gain further knowledge about the therapeutic management of the malignancy [53]. For instance, Agarwal et al. investigated the characteristics of pleomorphic liposarcoma of the head and neck and noted the importance of adjuvant therapy and negative resection margins in avoiding recurrent disease in a one-year follow-up [53]. Halevi PD et al. reported an unfavorable outcome in the case of a primary pleomorphic liposarcoma arising within the thoracic epidural space in a 70-year-old male patient who presented with lower extremities weakness and back pain [54]. The patient underwent surgical excision and received radiotherapy, but the lesion recurred 3 months after the surgery [54]. Later on, he developed metastases and succumbed to the disease one year later [54]. Researchers suggest that pleomorphic liposarcoma should be taken into consideration as a differential diagnosis for spinal tumor masses, although it is an exceptionally rare finding [54]. As mentioned earlier, immunohistochemical study is of limited use in diagnosing mesenchymal neoplasms within the heterogeneous group of pleomorphic sarcomas [52]. However, ancillary studies can be useful in distinguishing pleomorphic sarcomas from secondary tumors, as the lesions may be histologically similar to metastatic carcinoma or melanoma [52]. In selected cases, the immunohistochemical staining panel includes pancytokeratin, SOX10, and MelanA, while the expression pattern of p53 and INI 1 can also be interrogated [54]. In addition, immunohistochemical expression of MDM2 and CDK4 should be carried out, to rule out pleomorphic dedifferentiated liposarcoma, especially in tumors located within the retroperitoneum [52,55]. Al-Attar et al. reported a case of pleomorphic liposarcoma of the gastrointestinal tract in a 71-year-old patient with a history of rectal adenocarcinoma [56]. The lesion exhibited epithelioid cells with intracytoplasmic fatty droplets and high mitotic activity, which was initially interpreted as GIST, but expression of CD117 and DOG1 was absent and the lesion exhibited strong, diffuse positive p53 expression suggestive of a mutant phenotype [56,57]. Considering the rarity of pleomorphic liposarcoma and the reported peculiar locations of this malignancy, researchers acknowledge the significance of judiciously investigating the histopathological and immunohistochemical features of the tumors [56,57].

Myxoid pleomorphic liposarcoma is an uncommon, newly described liposarcoma variant, showing a propensity for mediastinum and usually affecting young patients and children [47,58]. This soft tissue neoplasm with a troublesome location typically shows aggressive behavior and a tendency for local recurrence [58]. Al-Kindi et al. reported a case of a giant mediastinal myxoid pleomorphic liposarcoma in an 18-year-old girl who underwent surgical excision followed by oncological treatment [47]. Upon histopathological examination, the tumor showed myxoid stroma encompassing spindle and stellate cells, as well as pleomorphic lipoblasts [47]. Immunohistochemical analysis revealed S100 positivity within the neoplastic cells [47]. Studies suggest that myxoid pleomorphic liposarcoma should be taken into consideration in the differential diagnosis of soft tissue tumors of the mediastinum in children and even infants [58]. As an example, Gami et al. reported the case of a 12-month-old infant presenting with respiratory distress who was finally diagnosed with myxoid pleomorphic liposarcoma [58]. In this patient’s case, the CT scan revealed a large solid tumor mass occupying the left hemithorax, and surgical resection was carried out [58]. The neoplastic proliferation disclosed pleomorphic multivacuolated lipoblasts and myxoid changes, as well as necrotic foci [58]. The tumor cells were immunoreactive for S100, CD 34, and p16 and negative for CDK4, SMA, and desmin, and fluorescent in situ hybridization was used for confirmation [52]. The patient later underwent chemotherapy to prevent disease recurrence [58,59]. Considering the rarity of this malignancy, its pathogenesis and histopathological features have been observed and reviewed throughout several studies. Researchers have distinguished similar histopathological features in lesions with different locations, although cases of myxoid pleomorphic liposarcoma occurring in peculiar sites have been reported [60]. As an example, Tan GZL et al. reported a case of myxoid pleomorphic liposarcoma of the orbit in a 12-year-old female patient with a gradually enlarging orbital mass causing proptosis of the globe [60]. The lesion was surgically excised and the patient received chemotherapy, with no distant metastasis discovered at the time of diagnosis [53]. Histopathological examination of the specimen revealed a liposarcoma with distinct myxoid and pleomorphic areas [60]. The myxoid areas displayed moderate cellularity and abundant myxoid matrix with occasional pulmonary-edema-like microcystic spaces, while the pleomorphic areas included bizarre cells with multivacuolated cytoplasm, suggestive of pleomorphic lipoblasts [60]. The neoplastic cells with adipocytic differentiation were highlighted using S100 and adipophilin immunohistochemical staining [60]. Mutant expression of p53 with a null pattern was also noted, and INI 1 expression was retained [60]. MDM2 expression was also interrogated, but the marker was negative within neoplastic cells, and in situ hybridization showed no evidence of DDIT3 rearrangement [60]. To achieve a better understanding of myxoid pleomorphic liposarcoma pathogenesis, researchers investigated the association of this malignancy with specific genetic abnormalities [60]. This pathological association is supported by the prevalence of pleomorphic liposarcoma in children and young patients [61]. In this matter, Zare SY et al. reported a case of pleomorphic liposarcoma in a 34-year-old patient with germline TP53 gene mutation and Li–Fraumeni syndrome [61]. The tumor presented as a well-demarcated, solid tumor mass located in the anterior chest wall, measuring 2.9 × 2.3 × 2 cm, and the histopathological aspect was characterized by low cellularity, myxoid stroma, and pleomorphic lipoblasts [61]. In situ hybridization detected no DDIT3 rearrangement or MDM2 amplification [61]. The authors acknowledge that this is the first case of myxoid pleomorphic liposarcoma associated with Li–Fraumeni syndrome and strongly recommend that further studies should be carried out regarding this condition [61]. Two more cases of myxoid pleomorphic liposarcoma affecting patients with Li–Fraumeni syndrome have been reported in the scientific literature so far. Francom et al. reported the case of a myxoid pleomorphic liposarcoma of the head and neck diagnosed as a second primary tumor in an 11-year-old boy with Li–Fraumeni syndrome, who had recently undergone chemotherapy for medulloblastoma [62]. Furthermore, Sinclair et al. reported the case of a 15-year-old female with Li–Fraumeni syndrome who developed a perineal mass, which was diagnosed as myxoid pleomorphic liposarcoma [63]. A retrospective review of malignancies encountered in children with Li–Fraumeni children has been carried out by Rodriguez et al. and included 20 children within a cohort of patients diagnosed with this condition [64]. Researchers reveal that 6 out of 27 malignancies reported in the analyzed group were sarcomas of the head and neck and were all demonstrated as rare entities—rhabdomyosarcoma, synovial sarcoma, and myxoid pleomorphic sarcoma [64]. The study identifies a propensity for head and neck involvement of tumors associated with Li–Fraumeni syndrome and recommends a multidisciplinary care team surveilling the affected patients [64].

## 4. Discussion

**Figure 1:**
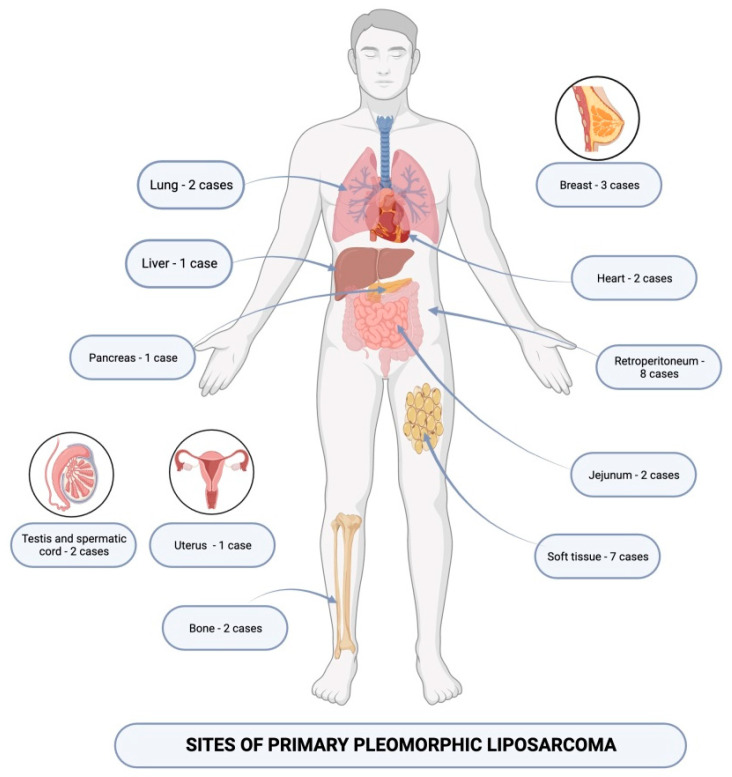
The primary location of pleomorphic liposarcomas.

Pleomorphic liposarcoma is the most uncommon liposarcoma of adult patients, and it can be associated with a severe prognosis and diagnostic difficulties [44,52,53,54]. We discovered 36 cases of pleomorphic liposarcomas reported during the last five years, between 2018 and 2023. Most of the tumors were located within the abdominal cavity and 8 out of 36 presented as large masses identified within the retroperitoneum or pelvic region [52]. Within the series reported by Wang L. et al., the group contained four men and two women, ranging from 46 to 82 years old, with a confirmed diagnosis of primary pleomorphic liposarcoma [52]. The lesions presented as rapidly growing tumors within the retroperitoneum, abdominal, and pelvic cavities and were identified upon computed tomography, then underwent surgical resection [52]. Histopathological examination of the specimens revealed the presence of bizarre mono or multinucleated giant lipoblasts, displaying heterotypic, hyperchromatic nuclei, and vacuolated cytoplasm [52]. Five out of six tumors had no invasion of the surrounding tissue, and none of the proliferations were associated with invasion of the lymph nodes or peripheral blood vessels [52]. However, in one case, the tumor mass infiltrated the fallopian tube, extending within its smooth muscle layer [52]. Immunohistochemical analysis showed positive CD 34 staining within the tumor cells in four cases, while expression of S-100 protein and CD 68 was identified in three cases [47]. Positive CD117 staining was noted in one of the lesions [52]. All tumors were negative for SMA and AE1/AE3 [47]. Local recurrence was reported in four out of the six patients [52]. This study highlights the aggressive clinical behavior of liposarcoma developing within the abdominal cavity, as lesions can be diagnosed in a locally advanced stage, and surgical resection can be difficult. Studies also acknowledge that pleomorphic liposarcoma of the retroperitoneum can be associated with acute severe systemic complications, apart from its locally infiltrative and distant metastatic potential [52,53,54,55,56,57,58,59,60,61,62,63,64,65]. As an example, Chen et al. reported a case of retroperitoneal pleomorphic liposarcoma associated with massive tumor embolism of the inferior vena cava and pulmonary arteries, discovered in a 54-year-old woman [65]. Local extension of pleomorphic liposarcoma developing within the retroperitoneum is one of the most frequent and concerning aspects of this malignant tumor. Involvement of the kidney was reported in two of the cases reported within the scientific literature that we interrogated [65]. El Haq et al. reported the case of an 84-year-old male patient who developed a pleomorphic liposarcoma with infiltration of the left flank and kidney, presenting with abdominal pain and hematuria [66]. The second most common location of pleomorphic liposarcoma is the soft tissue, with a total of seven reported cases. Concerning this pathology, studies show that distant metastases are rarely associated with liposarcoma of the soft tissue [66]. Therefore, special attention is required in such cases, as differential diagnosis may be necessary [66]. Ciliberti et al. reported the case of a 51-year-old man with a history of lung carcinoma who developed a pleomorphic liposarcoma of the shoulder girdle deep soft tissue and acquired hepatic metastases [67]. Considering the patient’s medical history, lung carcinoma metastasis was first taken into consideration, but a histopathological examination of the specimen revealed the presence of giant multinucleated lipoblasts with multivacuolated cytoplasm [67]. Immunohistochemical analysis revealed the expression of vimentin within the tumor cells, while MDM2 and Hep-Par1 were negative [67]. The authors underlined the importance of microscopic examination and ancillary studies for positive and differential diagnosis in patients with metastatic liposarcoma of the soft tissue [67]. Throughout our research, we identified one case of pleomorphic liposarcoma of the soft tissue that was exposed to neoadjuvant treatment. The case was reported by Zhang et al. in a 59-year-old woman who developed an unresectable liposarcoma of the deep soft tissue of the abdominal wall and received a combination of radiotherapy and angiogenesis inhibitor anlotinib [68]. The histopathological aspect of the tissue samples revealed the presence of pleomorphic epitheliod cells, occasionally associated with cytoplasmic vacuoles, but the immunohistochemical analysis was peculiar, because the neoplastic cells showed strong MDM2 expression [68]. However, no MDM2 or CDK4 rearrangements were detected, and the lesion was diagnosed as pleomorphic liposarcoma [68]. Examination of the surgical specimen also revealed significant changes related to neoadjuvant treatment, as the tumor volume was remarkedly reduced, and microscopic examination showed fibrotic areas and chronic inflammatory infiltrate, with no viable malignant cells [61]. The study infers that pleomorphic liposarcoma is regarded as a radiotherapy-resistant tumor; therefore, targeted chemotherapy such as angiogenesis inhibitors is required to obtain a complete pathological response [68]. Pleomorphic liposarcoma with a peculiar location was also investigated, as this lesion can reportedly occur within the viscera and bone. Liposarcomas of the testis and spermatic cord are exceptionally rare entities requiring special attention due to their high malignancy grade [69]. We discovered two pleomorphic liposarcoma of the testis and paratesticular region. In both of the cases reported, the lesions presented as large tumor masses, which were surgically excised, and the histopathological examination revealed the typical aspect, implying pleomorphic lipoblasts with multivacuolated cytoplasm [64,69,70]. According to the investigated articles, no immunohistochemical analysis was performed in addition to histopathological examination of the specimens, underlying the importance of a thorough microscopic evaluation of the samples. Surgical excision is regarded as the gold standard in the treatment of pleomorphic liposarcoma of the testis and paratesticular space [63]. However, researchers suggest that pre-operative radiotherapy should be taken into consideration in these malignant lesions, in order to reduce the tumor volume and obtain surgical resection margins [69]. Among pleomorphic liposarcomas with peculiar locations, malignant adipocytic tumors of the viscera require special attention [61,62,63,64,65,66,67,68,69,70,71,72]. We identified two primary pleomorphic liposarcomas of the lung, reported by Li B. et al. and Dey T. et al., both of them identified in male patients with no history of smoking or alcohol abuse [71,72]. The patients presented with shortness of breath and chest pain, and the tumors were described as large, hypodense masses identified using a CT scan [71,72]. The two patients received chemotherapy, followed by surgical resection; however, one of them developed a retroperitoneal metastasis 7 months after treatment [72]. Upon histopathological examination, both lesions were highly suggestive of pleomorphic liposarcoma [71,72]. An ancillary study revealed intense S100 positivity and a mutant p53 immunophenotype within the tumor cells of both lesions described above [72]. Primary pleomorphic liposarcoma of the lung is an exceedingly rare finding, accounting for less than 1% of pulmonary malignancies [72]. The main challenge regarding differential diagnosis of these lesions refers to metastatic liposarcoma; therefore, a thorough clinical examination and history taking should be performed, in addition to imagistic investigations and histopathological and immunohistochemical examination. Within the scientific literature that we investigated, two cardiac liposarcomas were found. Both lesions reported displayed characteristic locations, involving the interventricular septum and associated pericardial effusion [73,74]. Both lesions exhibited specific pleomorphic lipoblasts upon microscopic examination, while the ancillary studies performed revealed S100 positivity and a mutant p53 immunophenotype within the tumor cells [73,74]. Tan NY et al. underlined the importance of preoperative chemotherapy in primary cardiac liposarcoma, while Burt J.R. et al. described the benefit of eribulin therapy in the case of patients developing large tumors with pericardial involvement, in which case, surgical resection is undesirable [73,74]. In addition, a rare entity reported in the literature is pleomorphic liposarcoma of the breast. Throughout the investigated scientific literature, this proliferation has been reported in two cases, one female and one male patient, presenting as recurrent breast lump with locally aggressive behavior [75,76]. In both cases, modified radical mastectomy was performed, and microscopic examination revealed the typical pleomorphic liposarcoma aspect, with no lymph node metastases identified [75,76]. The authors underlined the importance of imaging studies in order to examine the tumor mass and assess its resectability [76]. To do this, mammography, ultrasonography, CT scan, and MRI can be used [68]. Although in both patients’ cases, the surgical resection margins were declared negative for tumor infiltration, both tumors recurred after surgery [76]. The aggressive clinical course and propensity for local recurrence are regarded as features strongly associated with the pleomorphic liposarcoma subtype, although sarcoma of the breast is incompletely understood due to its remarkable uncommonness [69]. Pleomorphic liposarcoma of the digestive tract is also a rare finding. However, we identified one liposarcoma of the pancreas and two liposarcoma involving the small intestine [77]. All the investigated lesions were surgically excised, and histopathological and immunohistochemical studies were carried out [54,73,74,75,76,77,78,79]. The tumor cells within the analyzed tumors showed positive S100 staining and were negative for CD34 and SMA [79]. Al-Attar et al. noted a mutant TP53 immunophenotype with hyperexpression [56]. None of the patients diagnosed with pleomorphic liposarcoma of the digestive tract reportedly developed metastases during the follow-up period [54,73,74,75,76,77,78,79]. Although liposarcoma is an exceptional finding in the female reproductive tract, clinicians should be aware of pleomorphic liposarcoma of the uterine corpus [79]. Valenciaga et al. reported a case of a 70-year-old patient who developed a liposarcoma of the uterus for which she received chemotherapy followed by a hysterectomy but was found with liver secondary determination 15 months after the surgery [79]. During the study, the patient received entrectinib as part of a clinical trial implying a multicenter global phase II basket study of this treatment for patients with metastatic solid tumors associated with genetic mutations such as NTRK, ROS 1, or ALK gene rearrangements [79]. Primary pleomorphic liposarcoma of the bone is also a rare clinical finding, with only two cases described within the past five years [80]. Tiemeier et al. described an intramedullary adipose tissue tumor mass located within the proximal tibia discovered in an 18-year-old male [80]. The lesion was treated with chemotherapy, comprising methotrexate, doxorubicin, and cisplatin, and later on, the tumor was surgically resected, and further reconstruction of the distal femur and proximal tibia was performed using endoprothesis [80]. Immunohistochemical analysis of the tumor revealed expression of FABP4/aP2, a marker of adipocytic differentiation in the neoplastic cells, while S100, CD34, MDM2, and SMA were negative [80]. The second case identified is that of a 20-year-old female who developed a pleomorphic liposarcoma of the femur, for which she underwent wide surgical resection and chemotherapy [81]. Immunohistochemical analysis of the tumor showed positive 100 staining, while expression of CD34, MDM2, and SMA was absent in the neoplastic cells [81]. No osteoblastic differentiation was disclosed, and the proliferation was negative for SATB2 and CD99 [81]. The patient succumbed to her disease 8 months after the initial diagnosis, as she had developed local recurrence and liver metastases [81]. Having considered all the reports discussed above, Figure 1 summarizes the possible primary locations of pleomorphic liposarcomas.

Furthermore, we also summarized the immunohistochemical markers used for diagnosing pleomorphic liposarcomas (Table 2).

## 5. Conclusions

Pleomorphic liposarcoma and myxoid pleomorphic liposarcoma are the rarest and most aggressive malignant tumors with adipocytic differentiation. Pleomorphic liposarcoma usually occurs within the retroperitoneum and somatic soft tissue of the extremities, but lesions with peculiar locations can also be encountered. Pleomorphic liposarcoma of the viscera can be a diagnostic challenge, considering the histopathological aspect of the proliferation, often mimicking a metastatic tumor. Microscopic examination is a key aspect in determining the correct diagnosis, due to the specific presence of malignant pleomorphic lipoblasts. Ancillary studies may be of limited use with regard to pleomorphic liposarcoma, and it is often limited to ruling out other malignancies with a specific immunohistochemical background, such as dedifferentiated liposarcoma. Pathogenesis and histopathological aspects of this liposarcoma variant are still incompletely understood, considering the scarce data available on this malignancy, due to the limited number of cases reported so far.

## Figures and Tables

**Table 1:** General characteristics of liposarcoma.

	Histopathology	Immunophenotype	Treatment
Well-Differentiated Liposarcoma	Lipogenic component with mature adipocytes of variable size Atypical spindle stromal cells	MDM2+, CDK+ DDIT3− S100+ CD34− p16+ p53 wildtype	Surgical resection with negative margins
Dedifferentiated Liposarcoma	Lipogenic component with an abrupt transition toward a non-lipogenic tumor area Necrosis [+/−]	MDM2+, CDK4+ DDIT3− S100+ CD34− p16+ p53 wildtype or mutant	Surgical resection Neoadjuvant radiotherapy Chemotherapy/targeted therapy
Myxoid Liposarcoma	Lipogenic areas Loose basophilic/myxoid stroma Necrosis [+/−] Atypical lipoblasts [+/−] Round cell component [+/−]	MDM2−, CDK4− DDIT3+ S100+ CD34− p16− p53 wildtype	Surgical resection Neoadjuvant or adjuvant radiotherapy Chemotherapy/targeted therapies
Pleomorphic Liposarcoma	High-grade undifferentiated sarcoma Pleomorphic lipoblasts Necrosis [+/−]	MDM2−, CDK4− DDIT3− S100+ CD34+ p16+ p53 mutant [hyperexpression]	Surgical resection or amputation Post-operative radiotherapy Chemotherapy
Myxoid Pleomorphic Liposarcoma	Myxoid liposarcoma-like areas Pleomorphic liposarcoma-like areas	MDM2−, CDK4− CD34+ p16+ p53 mutant [hyperexpression] Rb loss	Surgical resection with negative margins Benefits of radiotherapy and chemotherapy not well-established

**Table 2:** Immunophenotype of primary pleomorphic liposarcomas.

	S100	MDM2	CD 34	SMA	P53	P16
**Abdominal cavity**						
Wang et al. [6]	**+**		**+**	**-**		
**Soft tissue**						
Abe M. [82]	**+**	**-**	**-**			
Zhang C. [68]		**+**				
Piplani [83]	**+**		**-**			
Nautiyal [84]	**+**					
**Pulmonary**						
Li B. [71]	**+**	**+**	**-**		Hyper-expression/Mutant	**+**
Dey T. [72]	**+**	**-**			Hyper-expression/Mutant	
**Digestive tract**						
Yue [77]	**+**	**+**	**-**	**+**	Hyper-expression/Mutant	**+**
Meijide Santos G. [78]	**+**		**-**	**-**		
Al Attar M. [56]	**-**	**-**	**-**	**-**	Hyper-expression/Mutant	
**Bone**						
Farah S. [81]	**+**	**-**	**-**	**-**		
Tiemeier [80]			**-**	**-**		
**Cardiac**						
Tan NY. [74]	**+**				Hyper-expression/Mutant	
Burt JR. [73]	**+**				Hyper-expression/Mutant	
